# Comparative analysis of some evolutionary-based models in optimization of dam reservoirs operation

**DOI:** 10.1038/s41598-021-95159-4

**Published:** 2021-08-02

**Authors:** Mohammad Reza Sharifi, Saeid Akbarifard, Kourosh Qaderi, Mohamad Reza Madadi

**Affiliations:** 1 Department of Hydrology and Water Resources, Faculty of Water and Environmental Engineering, Shahid Chamran University of Ahvaz, Ahvaz, Iran; 2 Department of Water Engineering, Faculty of Agriculture, Shahid Bahonar University of Kerman, Kerman, Iran; 3 Department of Water Engineering, Faculty of Agriculture, University of Jiroft, Jiroft, Iran

**Keywords:** Hydrology, Engineering, Mathematics and computing

## Abstract

Deriving optimal operation policies for multi-reservoir systems is a complex engineering problem. It is necessary to employ a reliable technique to efficiently solving such complex problems. In this study, five recently-introduced robust evolutionary algorithms (EAs) of Harris hawks optimization algorithm (HHO), seagull optimization algorithm (SOA), sooty tern optimization algorithm (STOA), tunicate swarm algorithm (TSA) and moth swarm algorithm (MSA) were employed, for the first time, to optimal operation of Halilrood multi-reservoir system. This system includes three dams with parallel and series arrangements simultaneously. The results of mentioned algorithms were compared with two well-known methods of genetic algorithm (GA) and particle swarm optimization (PSO) algorithm. The objective function of the optimization model was defined as the minimization of total deficit over 223 months of reservoirs operation. Four performance criteria of reliability, resilience, vulnerability and sustainability were used to compare the algorithms’ efficiency in optimization of this multi-reservoir operation. It was observed that the MSA algorithm with the best value of objective function (6.96), the shortest CPU run-time (6738 s) and the fastest convergence rate (< 2000 iterations) was the superior algorithm, and the HHO algorithm placed in the next rank. The GA, and the PSO were placed in the middle ranks and the SOA, and the STOA placed in the lowest ranks. Furthermore, the comparison of utilized algorithms in terms of sustainability index indicated the higher performance of the MSA in generating the best operation scenarios for the Halilrood multi-reservoir system. The application of robust EAs, notably the MSA algorithm, to improve the operation policies of multi-reservoir systems is strongly recommended to water resources managers and decision-makers.

## Introduction

Water scarcity in many parts of the world makes the careful management of water resources necessary. Reservoirs are one of the most important elements of water management systems designed for storing and regularly releasing water to meet the downstream demands based on the operation policy. A reservoir operation policy includes a set of rules that assign water release in various operation conditions. Developing optimal operation policy for reservoirs is a complex and high challenging engineering problem, particularly for multi-reservoir systems. The main reasons for such complexity are the stochastic nature of the system inputs, the involvement of many decision variables and constraints, and the interfering operations between successive dams (in multi-reservoir systems).

Thus, the implementation of an appropriate reservoir operation policy needs a powerful optimization technique. Over the last few decades, several optimization methods have been proposed to solve such complex engineering problems. These methods can be classified into two main categories of classical methods, and evolutionary algorithms (EAs).

Classical methods such as linear programming (LP), non-linear programming (NLP), dynamic programming (DP), and stochastic dynamic programming (SDP), are old, imprecise and time-consuming techniques that often fail to provide reasonable solutions because they have several limitations, particularly for large scale multi-reservoir systems^1^. For example, the LP only can solve optimization problems with linear objective function and constraints, the NLP may get trap in a local optimum, especially in non-convex optimization problems, and the DP and the SDP suffer from the curse of dimensionality and state-space discretization^2,3^.

EAs are iterative search strategies in which a simulation process is repeated and a set of decision values is updated until the values satisfy a given stopping criteria, and, the optimal solutions are obtained by a decision-making process^4^. Over the last years, artificial intelligence-based EAs have been successfully employed to solve complex engineering problems^5–8^. Regarding their high capability in finding the optimal solutions, they have attracted the attention of researchers and water resources managers.

More recently, Qaderi et al.^9^ used a water cycle algorithm (WCA) for optimization of the monthly operation of Golestan and Voshmgir consecutive dams in Iran. They compared the results of the WCA with the genetic algorithm (GA), the harmony search algorithm (HS), the particle swarm optimization (PSO), and the imperialist competitive algorithm (ICA). It was found that the WCA was superior to the other algorithms in achieving the global optimum solutions. Mao et al.^10^ successfully employed the shuffled complex evolution (SCE) coupled with the stochastic ranking for reservoir scheduling problems. Ahmadebrahimpour^11^ used the wolf search algorithm (WSA) to optimize the operation of a four-reservoir system and a single hydropower system in Iran. He reported the supremacy of the WSA to the GA. Mohammadi et al.^12^ combined the whale optimization algorithm (WOA) with the GA to produce a hybrid whale-genetic algorithm with higher precision and convergence rate. They successfully applied the developed model to optimize the operation of two multi-reservoir benchmark systems. Zarei et al.^13^ integrated the bat algorithm (BA) and the PSO with the game theory for optimal operation of Shahid Dam Reservoir in Iran and reported the better convergence rate and higher reliability of the developed algorithm. Myo Lin et al.^14^ proposed a multi-objective model predictive control (MOMPC) for real-time operation of a multi-reservoir system in Myanmar. Chen et al.^15^ used the PSO with an adaptive random inertia weight strategy to optimize Panjiakou Reservoir system operation. The results indicated the higher efficiency of the developed model compared to the GA, the conventional PSO, and the improved versions of PSO. Shaikh^16^ utilized five different artificial neural network (ANN) models for optimal operation of Damodar Valley multi-purpose multi-reservoir system in India and documented the high capability of ANN models in this complex problem. Sharifi et al.^17^ developed a fitness-distance-balance moth swarm algorithm for optimization of cascade hydropower reservoirs operation. They found that the developed model could successfully increase the hydropower generation by 59.5% compared to the actual generation of energy over a 180-months operation period.

Recently, five robust EAs of tunicate swarm algorithm, TSA^18^, Harris hawks optimization algorithm, HHO^19^, seagull optimization algorithm, SOA^20^, sooty tern optimization algorithm, STOA^21^, and moth swarm algorithm, MSA^22^, were introduced and successfully employed in solving several real-world complex engineering problems. These algorithms showed very promising and competitive results compared to several well-known metaheuristics. They demonstrated many advantages such as the high convergence rate, the high capability in reaching the optimal values for large-scale problems, the minimal time consumption, the minimal computational cost in terms of CPU usage, and several other superiorities.

In the present study, we developed these recently introduced EAs to optimize the operation of Halilrood large-scale multi-reservoir system. To the best of the authors’ knowledge, this is the first application of these algorithms in optimization of water resources systems. To evaluate the efficiency of these algorithms, their results were compared with two popular algorithms of GA and PSO. In addition, four statistical metrics were employed to assess the algorithms’ performance.

## Methodology

### Optimization algorithms

Here a brief introduction to the utilized algorithms is presented. More mathematical details of each algorithm are referred to the original works. All the studied EAs were coded and run in the MATLAB R(2014)a platform.

#### TSA algorithm

Tunicate Swarm Algorithm (TSA), recently proposed by Kaur et al.^18^, is a new bio-inspired-based EA that is inspired by the jet propulsion and swarm behaviors of tunicates during the navigation and foraging process in the depth of ocean. Tunicate has the ability to find the location of food sources in the sea. Two behaviors of tunicate, jet propulsion and swarm intelligence, are employed for finding the food source. To mathematically model the jet propulsion behavior, a tunicate should satisfy three conditions, including avoiding the conflicts between search agents, the movement towards the position of the best search agent, and remaining close to the best search agent. The swarm behavior will update the positions of other search agents about the best optimal solution. The details of mathematical modeling of these behaviors were documented by Kaur et al.^18^.

**Figure 1:**
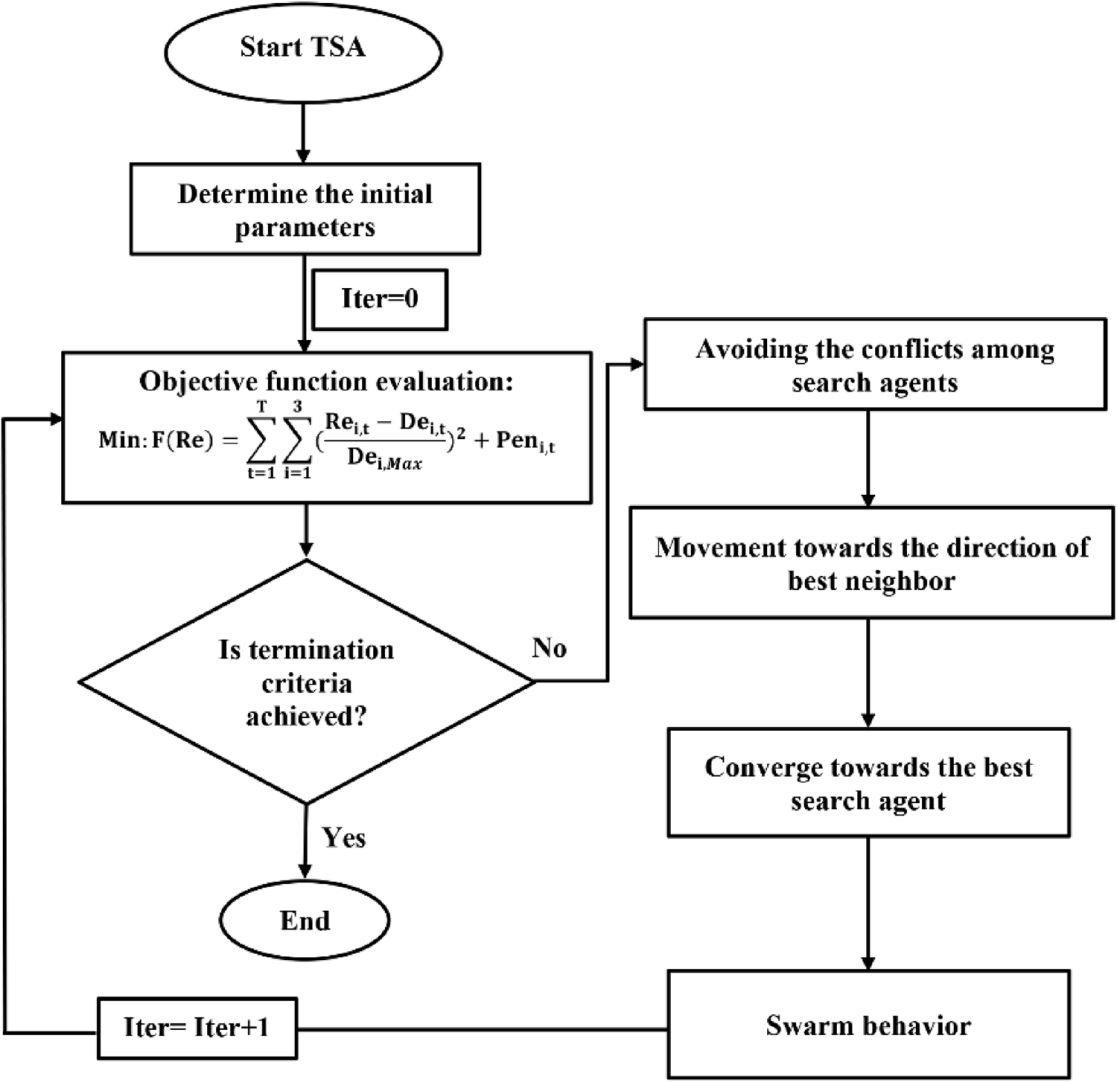
Flowchart of TSA algorithm in the optimal operation of Halilrood multi-reservoirs system.

In this study, we developed this algorithm to optimize the operation of Halilrood multi-reservoirs system. Figure 1 illustrates the phases of developed TSA in the optimization process.

#### HHO algorithm

The Harris Hawks Optimization (HHO) algorithm, proposed by Heidari et al.^19^, was inspired by the collaborative behavior and chasing style of Harris’ hawks in nature. The main strategy of Harris’ hawks to capture a prey is called “surprise pounce” or “seven kills” tactic. Harris’ hawks can conduct various chasing styles depending on the circumstances and escaping patterns of a prey. A switching strategy occurs when the best hawk stoops at the prey and gets lost, and the chase will be continued by one of the party members. The main advantage of these collaborative tactics is that the Harris’ hawks can pursue the detected prey to exhaustion, which increases its vulnerability. Besides, by perplexing the escaping prey, it loses its defensive capabilities and eventually, it gets caught by the most powerful and experienced hawks who share the captured prey with other party members. More details about this algorithm can be found in Heidari et al.^19^.

**Figure 2:**
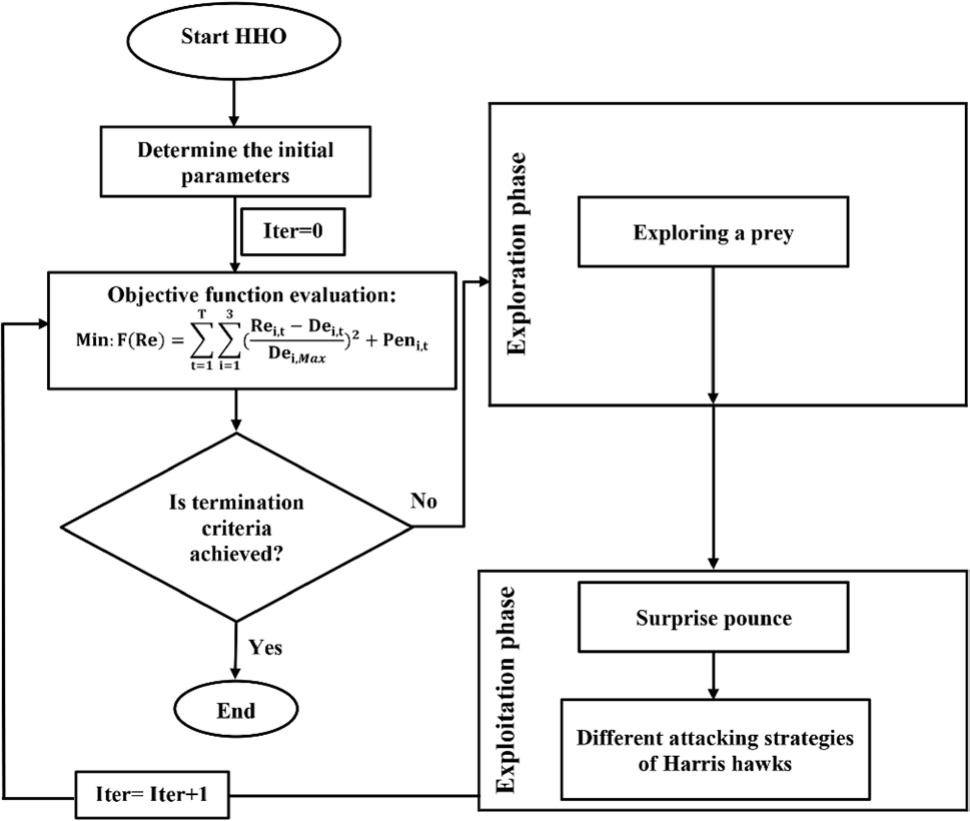
Flowchart of HHO algorithm in the optimal operation of Halilrood multi-reservoirs system.

The HHO is a population-based, gradient-free optimization technique; hence, it can be applied to any optimization problem subject to a proper formulation. Accordingly, we developed this algorithm for optimization of Halilrood multi-reservoir systems’ operation. Figure 2 shows the flowchart of HHO algorithm in the optimal operation of this problem.

#### SOA algorithm

Seagull optimization algorithm (SOA), proposed by Dhiman and Kumar^20^, stimulates the migration and attacking behaviors of a seagull in nature. Seagulls, with the scientific name of Laridae, are sea birds that are very intelligent. They use bread crumbs to attract fish and produce rain-like sounds with their feet to attract earthworms hidden under the ground. Generally, seagulls live in colonies. The most important behavior of the seagulls is their migrating and attacking behaviors. During migration, seagulls travel in a group. In the group, seagulls can travel towards the direction of the best survival fittest seagull. Therefore, according to the fittest seagull, other seagulls can update their initial positions. More details of the SOA can be found in Dhiman and Kumar^20^.

**Figure 3:**
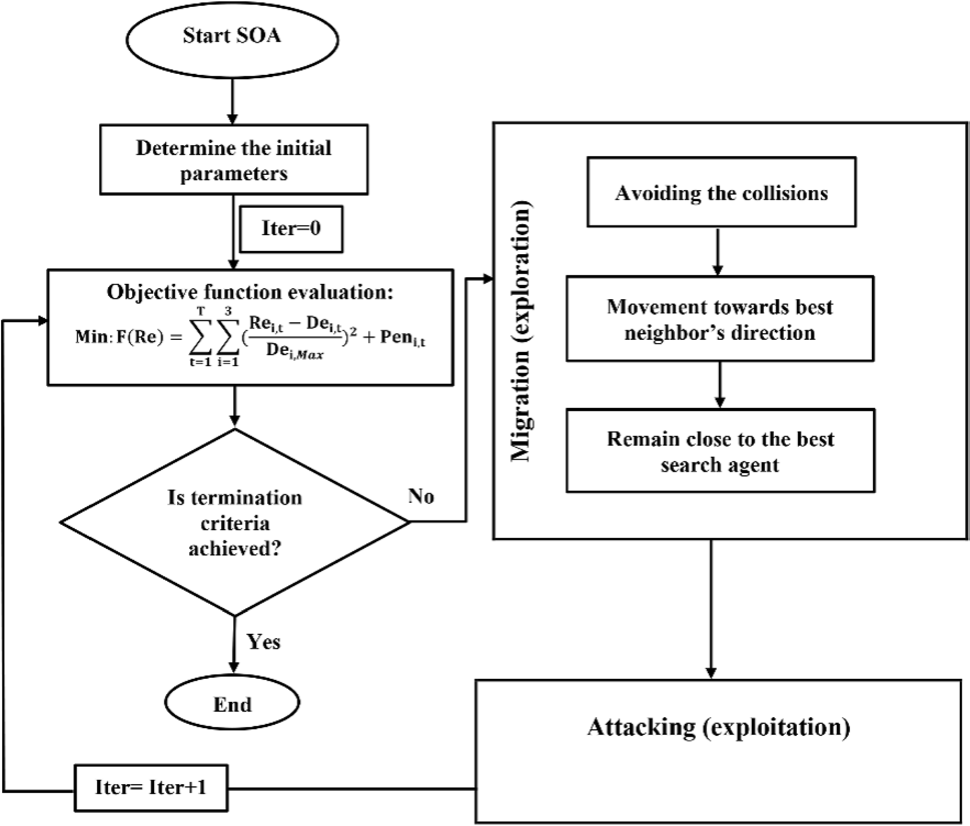
Flowchart of SOA algorithm in the optimal operation of Halilrood multi-reservoirs system.

Figure 3 demonstrates the flowchart of the SOA algorithm, including the migration and attacking phases, for the optimal operation of Halilrood multi-reservoirs system.

#### STOA algorithm

**Figure 4:**
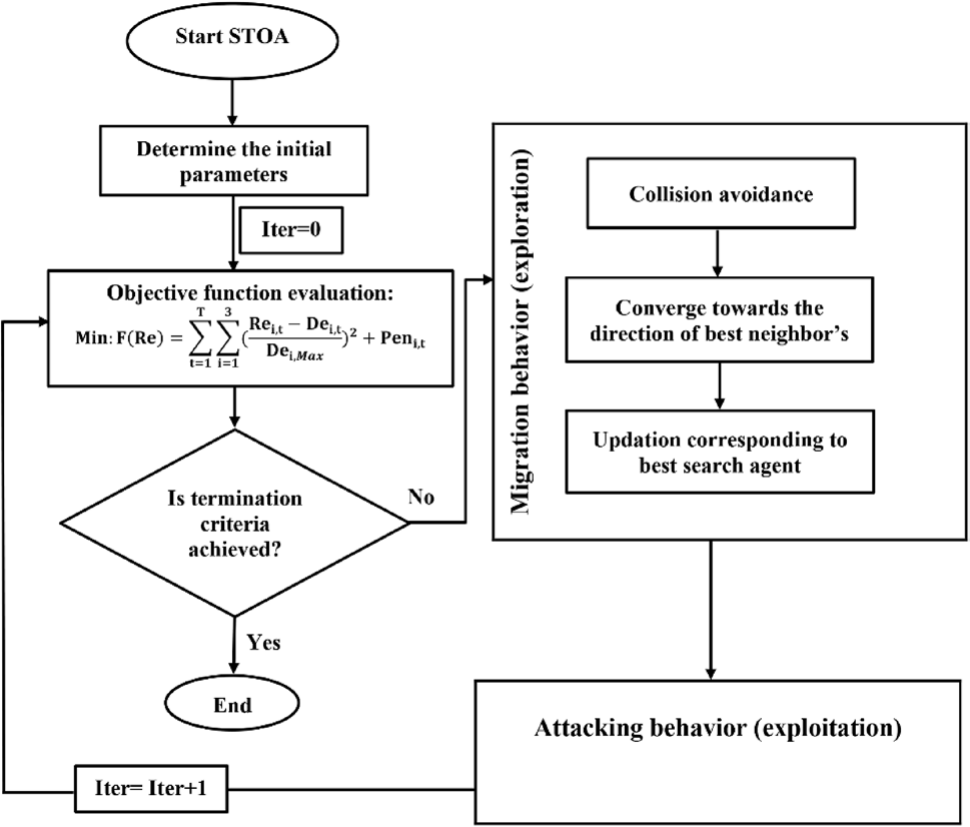
Flowchart of STOA algorithm in the optimal operation of Halilrood multi-reservoirs system.

Sooty Tern Optimization Algorithm (STOA), recently proposed by Dhiman and Kaur^21^, is a new evolutionary algorithm inspired by the behaviors of sooty tern in nature. Sooty terns, scientifically called *Onychoprion fuscatus*, are omnivorous birds that eat reptiles, insects, earthworms, fish, amphibians, etc. They live in colonies with a unique migrating and attacking behavior. Their migration is in group with a given distance between every two sooty terns to avoid collisions. In a group, sooty terns can travel towards the direction of best survival fittest sooty tern. Based on the fittest sooty tern, other sooty terns can update their initial positions. The migration and attacking steps are considered as exploitation and exploration phases in a given search space. In this study, the STOA was developed to optimize the operation of Halilrood multi-reservoirs system, as demonstrated in Fig. 4.

#### MSA algorithm

Moth swarm algorithm (MSA), proposed by Mohamed et al.^22^, was inspired by the behavior of moths in the nature. The moths try to hide from predators during the day, while looking for food resources at night with a celestial navigation technique. They fly in a straight line over a long distance by steering their locomotion at a steady angle relative to moonlight as the celestial far-distant point light. In the MSA, the possible solution is represented by the position of a light source, and the quality of this solution is considered the luminescence intensity of the light source. Three groups of moths (pathfinder, prospectors, and onlookers) are considered in the MSA. Pathfinders are capable of finding the best position over the optimization space with First-In, Last-Out principle to guide the movement of the main swarm. Prospectors tend to wander into a random spiral path nearby the light sources, which have been marked by the pathfinders. Onlookers drift directly toward the best global solution (moonlight), which has been achieved by prospectors’ moths. In each iteration at MSA, each moth enters the problem to find the corresponding luminescence intensity of the light source. The best fitness in the population is considered as the position of pathfinder guiding for the next iteration. Thus, the second and third best groups are called prospectors and onlookers, respectively. The MSA algorithm is performed through three phases of initialization, reconnaissance, and transverse orientation^22^.

**Figure 5:**
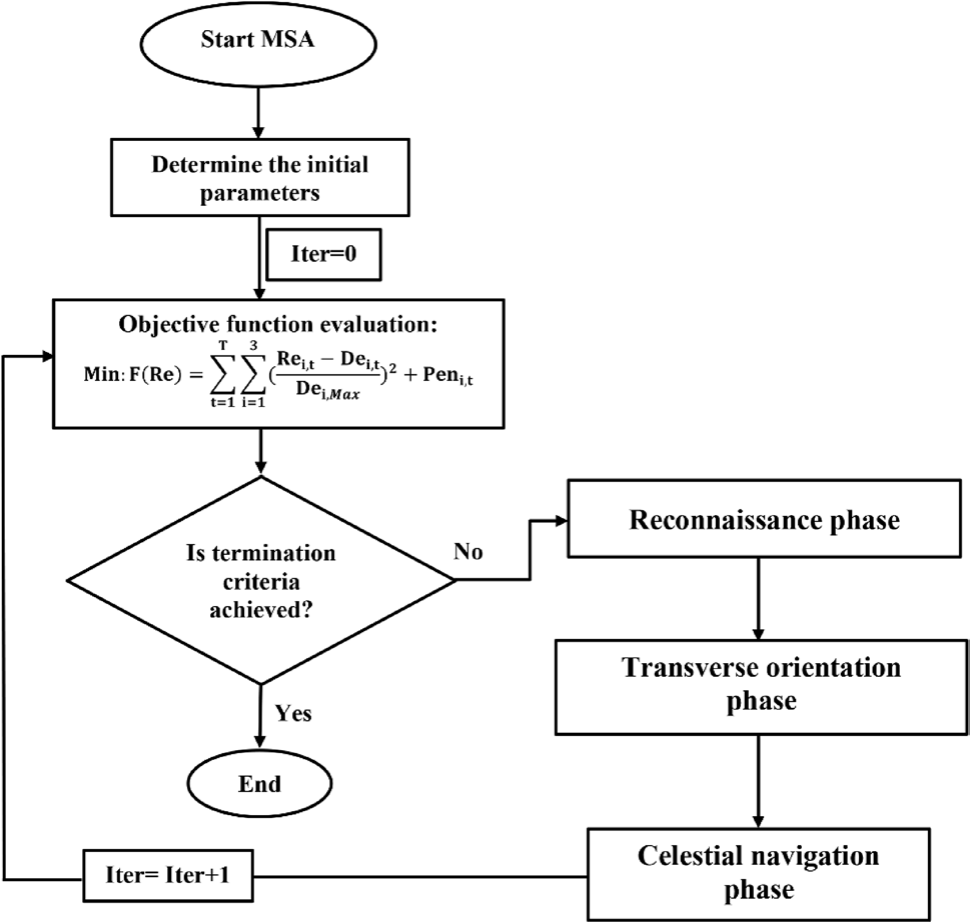
Flowchart of MSA algorithm in the optimal operation of Halilrood multi-reservoirs system.

Figure 5 shows phases of the MSA algorithm in the optimal operation of Halilrood multi-reservoirs system.

#### PSO algorithm

The particle swarm optimization (PSO), proposed by Kennedy and Eberhart^23^, is a metaheuristic algorithm inspired by the swarm behavior of flocks of birds or schools of fish in nature. These swarms follow a cooperative way to find food, and each member in the swarms keeps changing the search pattern according to the learning experiences of its own and other members. While searching for food, the birds are either scattered or go together before they locate the place where they can find the food. While the birds are searching for food from one place to another, there is always a bird that can smell the food very well, that is, the bird is perceptible of the place where the food can be found, having the better food resource information. Because they are continuously exchanging information about the food place, the birds will eventually flock to the place where better food can be found.

**Figure 6:**
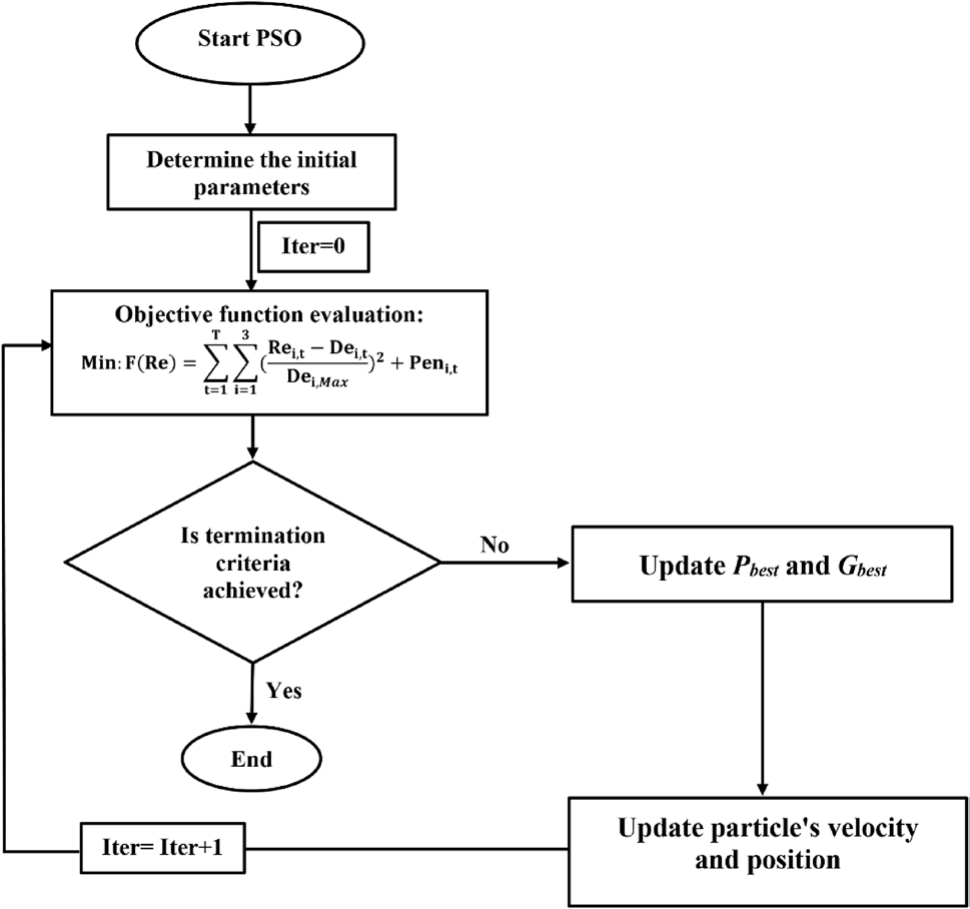
Flowchart of PSO algorithm in the optimal operation of Halilrood multi-reservoirs system.

In the PSO algorithm, solution swam is equal to the bird swarm, the birds’ moving from one place to another is equal to the development of the solution swarm, good information is equal to the most optimist solution, and the food resource is equal to the most optimist solution during the whole course. The position of each particle in the swarm is affected both by the most optimist position during its movement and the position of the most optimist particle in its surrounding. In other word, the movement of each particle is identified in two phases of exploration (global search) and exploitation (local search). In the exploration phase, particle fly across the whole search space to find a limited region containing the global optimum. After the region containing the global optimum has been found, the exploitation phase is begun. The position of each particle is corrected by making small movements in the neighborhood of the global optimum. By adopting the correct sequence of these two phases, it is possible to lead particles towards the global optimum. Figure 6 shows the flowchart of the PSO algorithm in the optimal operation of Halilrood multi-reservoir system.

#### GA algorithm

**Figure 7:**
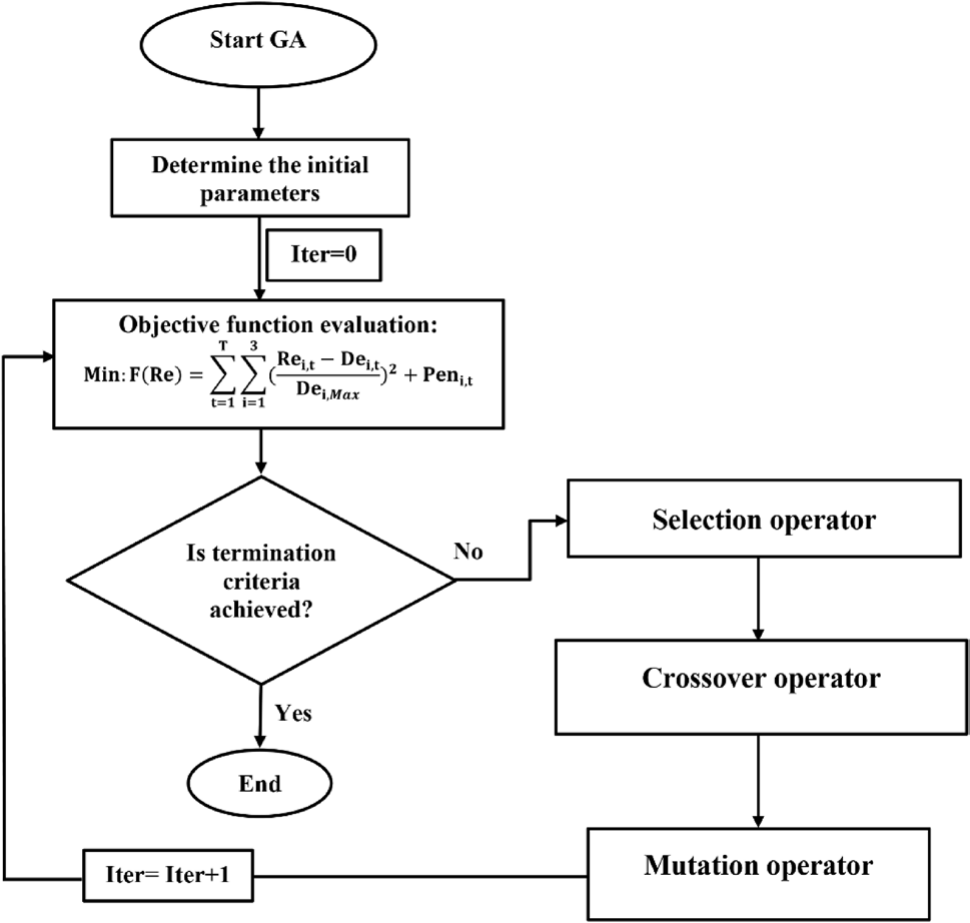
Flowchart of GA algorithm in the optimal operation of Halilrood multi-reservoirs system.

The Genetic Algorithm (GA), proposed by Holland^24^, is one of the most popular EAs that was inspired by Charles Darwin's theory of natural evolution. This algorithm reflects the process of natural selection where the fittest individuals are selected for reproduction in order to produce offspring of the next generation. GA algorithm starts with an initial set of random solutions, called population. Each solution in the population is called a chromosome, which represents a point in the search space. The chromosomes evolve through successive iterations, called generations. During each generation, the chromosomes are evaluated using some measures of fitness. The fitter the chromosomes, the higher the probabilities of being selected to perform the genetic operations, including crossover and mutation. In the crossover phase, the GA attempts to exchange portions of two parents, that is, two chromosomes in the population to generate an offspring. The crossover operation speeds up the process to reach better solutions. In the mutation phase, the mutation operation maintains the diversity in the population to avoid being trapped in a local optimum. A new generation is formed by selecting some parents and some offspring according to their fitness values, and by rejecting others to keep the population size constant. After the predetermined number of generations is performed, the algorithm converges to the best chromosome, which hopefully represents the optimal solution or maybe a near-optimal solution of the problem. Figure 7 shows the flowchart of the GA algorithm in the optimal operation of Halilrood multi-reservoir system problem.

### Simulation–optimization model

**Figure 8:**
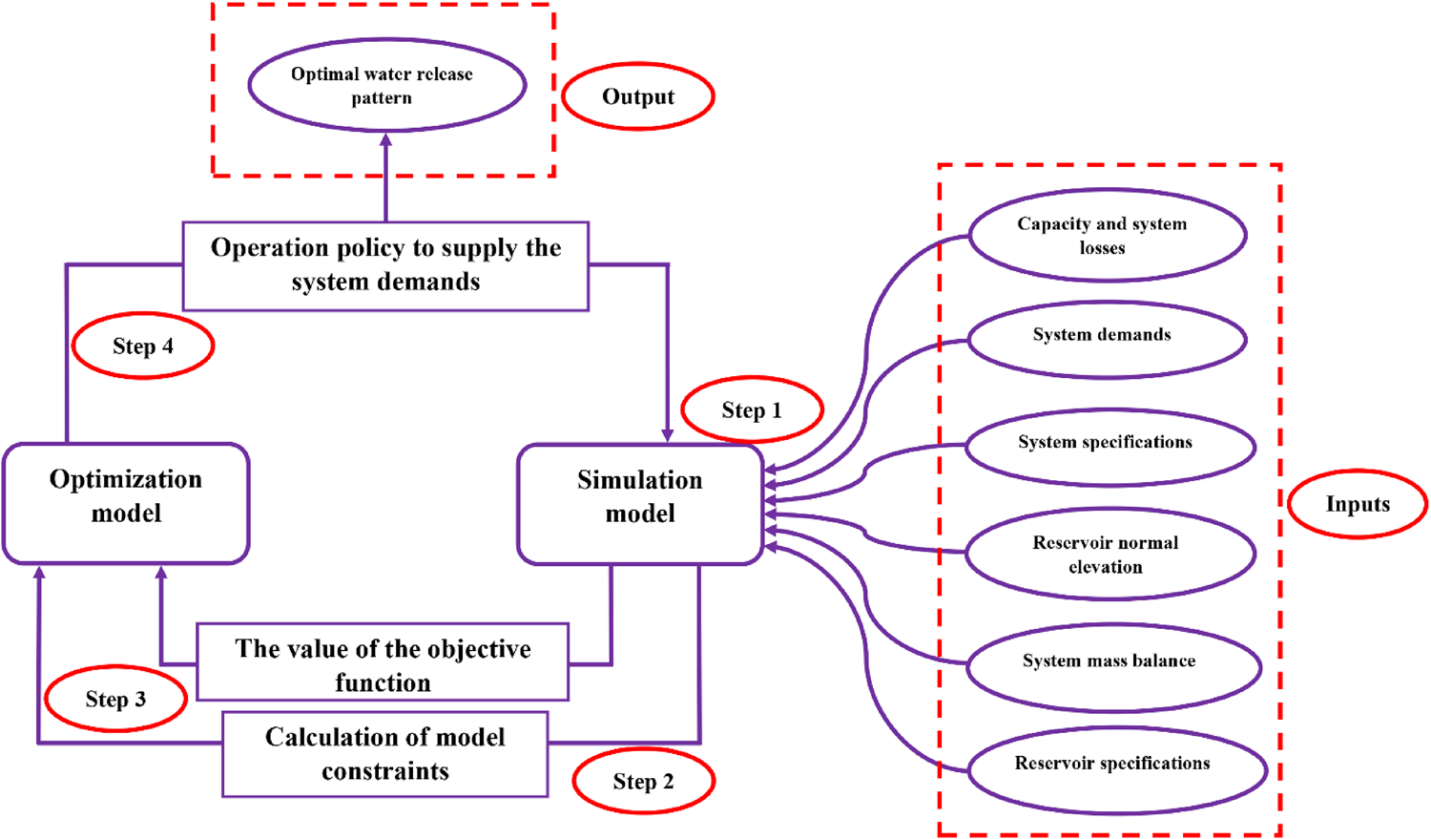
Conceptual model of simulation–optimization of operation of Halilrood reservoir system.

The conceptual scheme of the simulation–optimization model was demonstrated by Fig. 8. As seen, this is an integrated and comprehensive model because all the influencing parameters on the problem have been accounted. This optimization-simulation model was developed in a way that the best operational policies for Halilrood multi-reservoir system are achieved.

The objective function of the optimization model was defined as the minimization of the total deficit over the operational period. The deficit means the difference between the amount of release from reservoirs and the amount of the downstream demands (drinking, agriculture, environment and industry demands). The greater the amount of deficit, then the greater the failure of the system. Release from each reservoir in each period was considered as a decision variable, and the storage capacity and the inflow to the reservoir were the state variables. The planning horizon was 223 months from October, 2000 to April, 2019. Therefore, the utilized algorithms have 669 variables (223 × 3) in the model of operation of Halilrood multi-reservoir system, making it a large-scale problem. The term “large-scale” is related to the dimension of the problem (the number of decision variables). The model constraints were similar for all three reservoirs. The criterion for measuring solutions was comparing them with the downstream demands and the best values of storage in different months. Inputs of the model include river flow discharge, evaporation rate, precipitation height (rainfall or snow depth measured by a rain gauge), and amount of demands per month. The objective function of the multi-reservoir system is expressed as Eq. (1).

1 $$Minimize \; \; F\left(Re\right)=\sum_{t=1}^{T}\sum_{i=1}^{3}{\left(\frac{{Re}_{i,t}-{De}_{i,t}}{{De}_{i,Max}}\right)}^{2}+Penalty$$

where, *F(Re)* is the value of the objective function (deficit), *Re*_*i,t*_ is the release from the *ith* reservoir during period *t* (MCM), *De*_*i,t*_ is the downstream demands of the *ith* reservoir during period *t* (MCM), *De*_*i,Max*_ is the maximum downstream demand of the *ith* reservoir in the whole operation period (MCM), *T* is the total number of operation periods (month), *t* is the index for simulation periods, *i* is the reservoir index, which *i* is 1 for Baft reservoir, *i* = 2 for Safarood reservoir, and *i* = 3 for Jiroft reservoir. *Penalty* denotes the penalty function on storage deviations outside the feasible region in *ith* reservoir in month *t.* Constraints of the multi-reservoir system are expressed by Eqs. (2)–(10).

(I) The constraint on the volume of overflow and evaporation losses:
  2 $${Sp}_{i,t}=\left\{\begin{array}{ll}{S}_{i,t}-{S}_{max i}+{S}_{\mathit{min}i} & \quad if \; {S}_{i,t}>({S}_{\mathit{max}i}-{S}_{\mathit{min}i})\\ 0 & \quad if \; {S}_{i,t}\le ({S}_{\mathit{max}i}-{S}_{\mathit{min}i})\end{array}\right.$$
  3 $${Loss}_{i,t}=\frac{\left[{A}_{i,t}\times \left(\frac{{Ev}_{i,t}}{1000}\right)\right]}{1000}$$
  4 $${A}_{i,t}={a}_{i}+{b}_{i}\times {S}_{i,t}+{c}_{i}\times {S}_{i,t}^{2}$$
(II) mass balance between inflow and outflow regarding the reservoir storage capacity:
  5 $${S}_{1,t+1}={S}_{1,t}+{Q}_{1,t}-{Re}_{1,t}-{Sp}_{1,t}-{Loss}_{1,t}$$
  6 $${S}_{2,t+1}={S}_{2,t}+{Q}_{2,t}-{Re}_{2,t}-{Sp}_{2,t}-{Loss}_{2,t}$$
  7 $${\mathrm{S}}_{3,\mathrm{t}+1}={\mathrm{S}}_{3,\mathrm{t}}+{\mathrm{Q}}_{3,\mathrm{t}}+{\mathrm{Sp}}_{1,\mathrm{t}}+{\mathrm{Sp}}_{2,\mathrm{t}}+{\mathrm{Dez}}_{1,\mathrm{t}}+{\mathrm{Dez}}_{2,\mathrm{t}}-{\mathrm{Re}}_{3,\mathrm{t}}-{\mathrm{Sp}}_{3,\mathrm{t}}-{\mathrm{Loss}}_{3,\mathrm{t}}$$
(III) Constraints of decision variables
  8 $${S}_{\mathit{min}i}\le {S}_{i,t}\le {S}_{\mathit{max}i}$$
  9 $${Re}_{\mathit{min}i,t}\le {Re}_{i,t}\le {Re}_{\mathit{max}i,t}$$
(IV) Penalty function of storage deviations outside the feasible region
  10 $$Penalty=\left\{\begin{array}{ll}\sum_{t=1}^{T}\sum_{i=1}^{3}{(\frac{{S}_{i,t}-{S}_{\mathit{min}i}}{{S}_{\mathit{min}i}})}^{2} & \quad if \; {S}_{i,t}<{S}_{\mathit{min}i} \\ \sum_{t=1}^{T}\sum_{i=1}^{3}{(\frac{{S}_{i,t}-{S}_{\mathit{max}i}}{{S}_{\mathit{max}i}})}^{2} & \quad if \; {S}_{i,t}>{S}_{\mathit{max}i} \\ 0 & \quad if \; {S}_{i,t}\ge {S}_{\mathit{min}i} \; and \; {S}_{i,t}\le {S}_{\mathit{max}i}\end{array}\right.$$
  where, *Sp*_*i,t*_ is the overflow from the *ith* reservoir during period *t* (MCM), *S*_*i,t*_ and *S*_*i,t*+*1*_ are the storages of the *ith* reservoir at the beginning and end of period *t* (MCM), *S*_*maxi*_ is the maximum storage in the *ith* reservoir during period *t*, *S*_*mini*_ is the minimum storage of the *ith* reservoir at the beginning of period *t*, *Q*_*i,t*_ is the inflow into the *ith* reservoir during period *t* (MCM), *Loss*_*i,t*_ is the evaporation loss from the *ith* reservoir surface during period *t* (MCM), *A*_*i,t*_ is the area of the *ith* reservoir during period *t* (KM^2^), *Ev*_*i,t*_ is the net evaporation (evaporation minus precipitation) from the *ith* reservoir during the period *t* (m), *a*_*i*_, *b*_*i*_ and *c*_*i*_ are the coefficients of storage-area relation for reservoir *i*. *Dez*_*i,t*_ is the environmental demand of *ith* reservoir during period *t* (MCM), *Re*_*maxi,t*_ and *Re*_*mini,t*_ are the maximum and minimum allowable release from the *ith* reservoir during period *t*, respectively.

### Performance indicators

Evaluating operation policies is the most important step in utilizing the optimization- simulation model. This is done by four indices of reliability, vulnerability, resiliency, and sustainability, which are formulated as follow:

(I) *Reliability* Reliability index refers to the percentage of periods, during which the system has completely supplied the existing demands. Equation (11) shows the formulation of this index^25^.
  11 $$Rel=\left(1-\frac{{NDe}_{f}}{T}\right)\times 100, \quad {ND}_{ef}=Number \; of \; \left({De}_{i}>{Re}_{i}\right)$$
  where *NDe*_*f*_ refers to the total number of failures occurred during the operation period, *De*_*t*_ refers to the demand in period *t*, *Re*_*t*_ is the release value of period *t*, *Rel* indicates the reliability of the system during the operation period. The higher the value of this index, the greater the reliability of the system.
(II) * Vulnerability* This index shows the magnitude of system failure, which is calculated from Eq. (12) ^25^.
  12 $$Val=max\left\{\frac{({De}_{t}-{Re}_{t})}{{De}_{t}}\right\}\times 100, \quad t=\mathrm{1,2},\dots ,t$$
  where *Val* refers to system vulnerability, *De*_*t*_ refers to demand in period *t*, *Re*_*t*_ is the release value in period *t*, and *T* indicates the total number of operating periods.
(III) * Resiliency* This index shows the system's capability to change the existing conditions. The likelihood that a system will return to normal after failure is called resiliency^25^. The value of this parameter can be calculated by the following equation:
  13 $$Res=\frac{\begin{array}{c}T\\ N\\ t=1\end{array}({Def}_{t+1}=0 | {Def}_{t}>0)}{\begin{array}{c}T\\ N\\ t=1\end{array}({Def}_{t}>0)}\times 100, \quad t=\mathrm{1,2},\dots ,T$$
  where $$\begin{array}{c}T\\ N\\ t=1\end{array}()$$ refers to the number of periods, in which the condition in parentheses has occurred, and *Def*_*t*_ refers to water deficit in period *t*.
(IV) * Sustainability index* This index is the summary of system performance criteria in an overall index to facilitate the comparison and decision-making between various scenarios based on reservoir performance indicators that are defined as follows^26^:
  14 $$SI={\left\{Rel\times Res\times (1-Vul)\right\}}^{1/3}$$

### Case study

**Figure 9:**
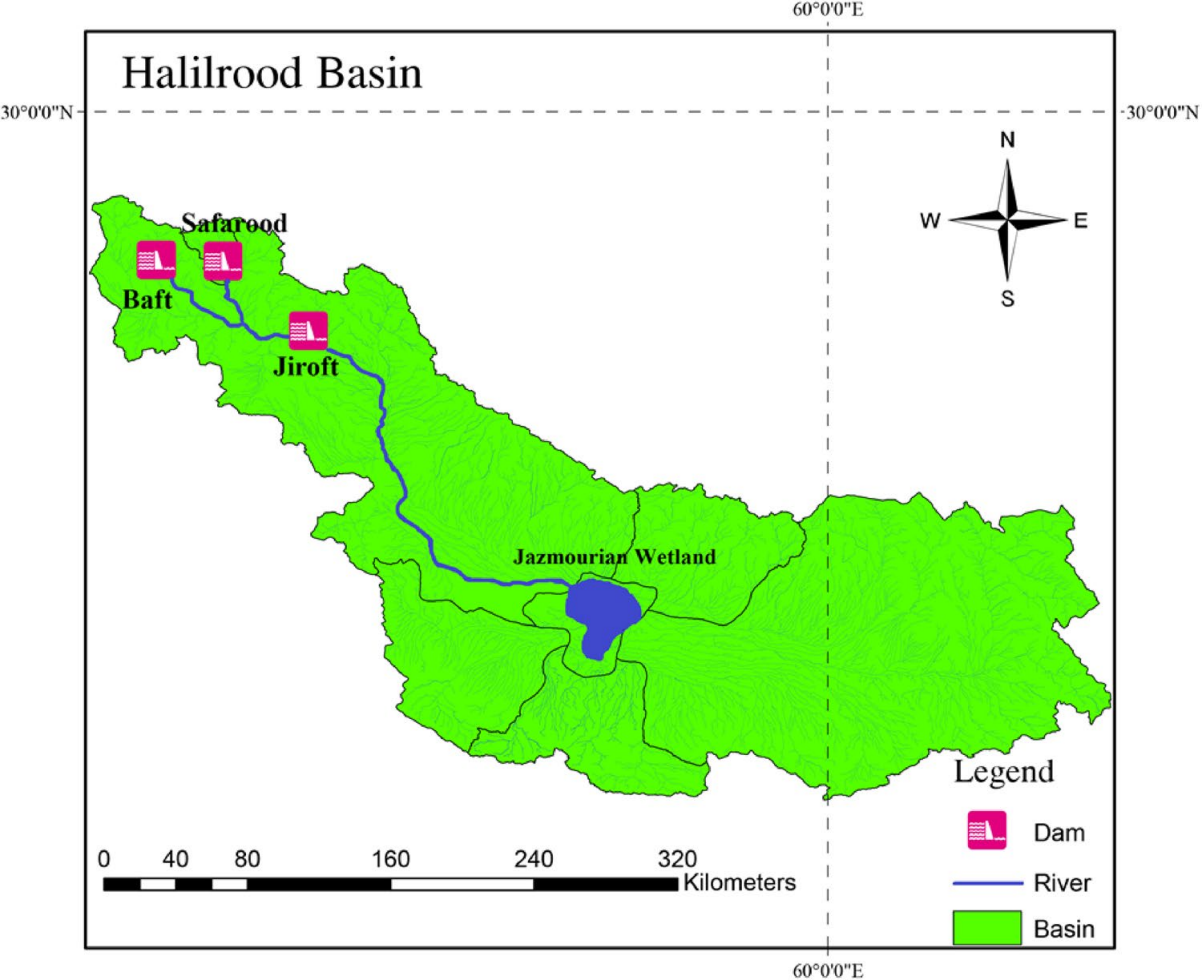
Location of Halilrood multi-reservoirs system. This figure was constructed by authors through version 10.2.2 of ArcGIS software (https://www.arcgis.com).

Halilrood is one of the main sub-basins of the large Hamoun-Jazmourian basin, which is located in the southeast of Iran between longitude 56°–51′ to 61° 30′ east and latitude 26° 18′ to 29° 30′ north. Its total area is 7224 km^2^ (Fig. 9).

**Figure 10:**
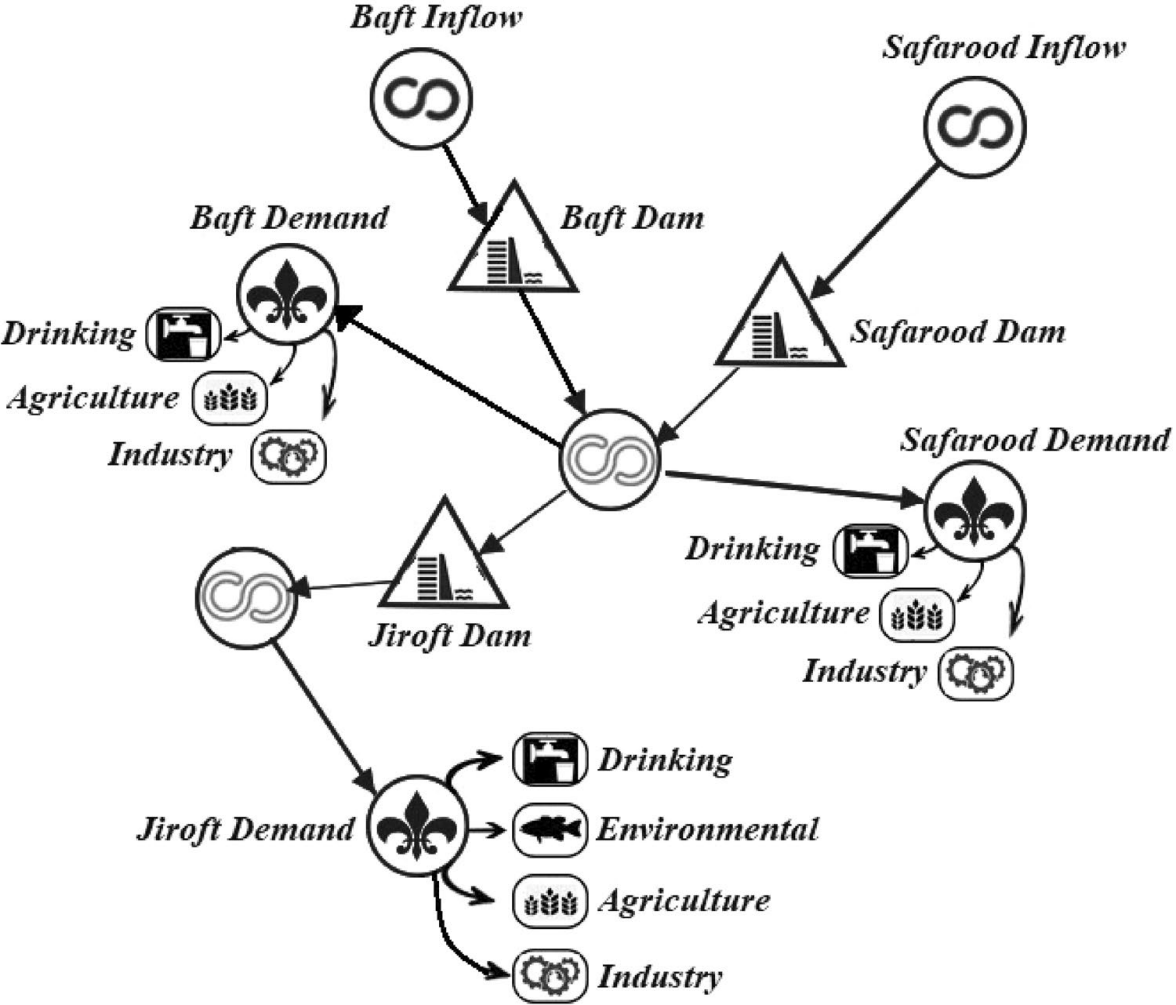
The schematic representation of Halilrood multi-purpose multi reservoir dams.

The range of elevation varies from more than 4000 m at the upstream to less than 500 m at the downstream. Halilrood is the major river in this basin which discharges to the Jazmorian Wetland at the end. Three large multi-purpose dams exist along this river tributaries, including (I) Baft earth dam with the purpose of water supply, (II) Jiroft concrete arch dam for water supply, electricity generation and flood control purposes, and (III) Safarood dam (under construction) for water supply purposes, which together act as a multi-reservoir system. Figure 10 demonstrates the schematic view of the Halilrood multi reservoir system.

The increasing water extraction from various uses and the lack of appropriate water allocation have imposed a significant impact on the downstream areas, especially on the Jazmorian Wetland, and caused serious conflicts in the region. If proper measures are not taken in the operation of existing reservoirs, the situation will get worse. One of the best strategies to overcome these issues is the optimization of the operation of this multi-reservoir system. In this study, five new robust evolutionary algorithms compared with two optimization algorithms of GA and PSO, were employed to derive the operation policies for Halilrood multi-reservoir system. The term “robust” refers to the algorithm with a set of features such as rapid convergence, time saving in long runs and uncertainty removal about the optimum solution.

## Results and discussion

As previously described, to derive the long-term operation policies of Halilrood multi-purpose multi-reservoir dams (including Baft, Safaroud, and Jiroft dams), a series of recently introduced robust evolutionary algorithms in addition to the well-known GA and PSO algorithms were developed in this study. Here, the results of these algorithms were compared together, and the best one was selected based on the values of four performance metrics.

A sensitivity analysis on the parameters of the utilized algorithms was done to obtain the best values for each parameter. Table 1 indicates the best values of parameters for Halilrood multi-reservoir system.

**Table 1:** Parameter setting values for algorithms.

Algorithms	Parameters	Values
# For all algorithm	Maximum iterations	20,000
	Number of variables	669
MSA	Number of search agents	300
	Number of pathfinders	20
HHO	Number of search agents	300
SOA	Search agents	300
	Control parameter (A)	[2,0]
	f_c_	2
STOA	Search agents	300
	C_f_	2
TSA	Search agents	300
	Parameter *P*_*min*_	1
	Parameter *P*_*m*ax_	4
GA	Number of genes	300
	Mutation rate	0.01
	Crossover rate	0.8
PSO	Population size	300
	C1	1.49
	C2	1.49

**Figure 11:**
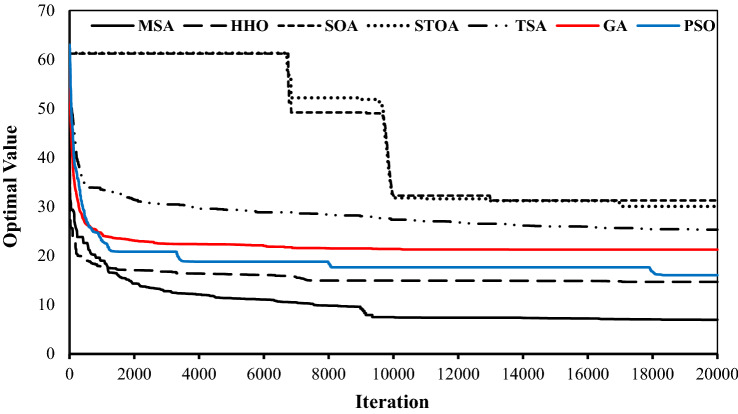
Convergence rate of utilized algorithms in operation of Halilrood multi-reservoirs system.

Figure 11 demonstrates the convergence rate of applied algorithms in reaching the optimum value for the operation of Halilrood multi-reservoir system. It indicates the rapid convergence rate of the MSA in comparison with the HHO, SOA, STOA and TSA.

As seen, the highest convergence rate belongs to the MSA and HHO algorithms, respectively, and the slowest convergence rate is seen at the STOA and SOA. If the rate of convergence is higher, then typically fewer iterations are needed to yield an optimum solution, and therefore, the computational costs decreases. Among the utilized algorithms, the MSA has performed better in this regard; it could approach to the optimal value before 2000 iterations. The more popular algorithms of GA and PSO were placed in the middle ranks among all.

The values of the objective function, the average CPU time, and the algorithms capability to meet the reservoirs downstream demands were presented in Table 2, based on PC with i7 CPU 1.8 GHz/16 GB RAM/2TB HDD. As shown, MSA could yield the best objective function (= 6.96) in the shortest CPU time (= 6738.56 s), while the corresponding values for the other utilized algorithms were not desirable. The MSA could supply 88.31%, 73.23% and 99.75% of downstream demands of Baft dam, Safarood dam and Jiroft dam, respectively, indicating the high performance of MSA in optimizing the studied multi-reservoir system. The next rank belongs to the HHO algorithm with the objective function of 14.68 and the CPU time of 10,223.4 s, which supplied 81.75%, 64.63%, and 98.26% of downstream demands of this multi-reservoir system with the same order.

**Table 2:** Objective function and deficit of reservoir demands resulting from the run of algorithms.

Algorithm	CPU time (s)	OF	Baft dam		Safarood dam		Jiroft dam	
			Deficit (MCM)	Supply (%)	Deficit (MCM)	Supply (%)	Deficit (MCM)	Supply (%)
MSA	6738.56	6.96	37.66	88.31	151.68	73.23	2.99	99.75
HHO	10,223.4	14.68	58.78	81.75	200.46	64.63	20.52	98.26
SOA	12,971.24	31.28	63.74	80.21	162.89	71.26	290.21	75.40
STOA	13,762.63	29.55	61.98	80.75	173.97	69.30	254.42	78.44
TSA	14,416.42	25.32	89.57	72.19	171.31	69.77	252.38	78.61
GA	11,781.62	21.26	58.32	81.89	185.31	67.30	177.76	84.93
PSO	9347.28	16.04	65.74	79.59	165.08	70.87	125.15	89.39

Table 3 shows the values of performance metrics of the reservoirs (reliability, resilience, vulnerability, and sustainability), resulting from the running of MSA, HHO, SOA, STOA, TSA, GA, and PSO algorithms in the long-term operation of the Halilrood multi-reservoir system.

**Table 3:** Performance evaluation criteria of the reservoirs in the operation of the Halilrood multi-reservoir system.

Demand	Algorithm	Reliability	Resilience	Vulnerability	Sustainability
Baft	MSA	73.99	34.91	24.97	57.87
	HHO	67.26	38.43	50.19	50.50
	SOA	53.81	19.18	96.64	15.14
	STOA	56.95	22.12	94.26	19.34
	TSA	40.81	23.08	96.88	14.33
	GA	61.88	24.22	76.20	32.92
	PSO	62.02	25.37	56.38	40.94
Safarood	MSA	49.35	29.77	63.97	37.55
	HHO	22.45	15.99	75.36	20.68
	SOA	32.74	14.41	75.54	22.60
	STOA	25.56	12.16	75.20	19.75
	TSA	32.29	15.28	75.66	22.90
	GA	22.42	15.70	75.36	20.54
	PSO	34.53	18.40	75.66	24.91
Jiroft	MSA	99.55	73.65	12.99	86.09
	HHO	92.83	49.79	27.34	69.51
	SOA	56.05	20.00	96.15	16.28
	STOA	63.68	21.00	93.38	20.69
	TSA	49.01	23.51	91.81	21.14
	GA	63.68	29.08	84.92	30.34
	PSO	62.78	36.67	36.33	52.72

As previously said, the sustainability criterion is an index for determining the performance of water resources systems. From Table 3, the sustainability criteria obtained by the MSA algorithm to supply the demand of Baft, Safarood, and Jiroft reservoirs are equal to 57.87, 37.55 and 86.09, respectively. This criterion is higher than the corresponding values in the new HHO (20.50, 20.68, 69.51), SOA (15.14, 22.60, 16.28), STOA (19.34, 19.75, 20.69) and TSA (14.33, 22.90, 21.14), GA (32.92, 20.54, 30.34), and PSO (40.94, 24.91, 52.72) algorithms. The comparison of these values indicates the high capability and accuracy of MSA algorithm in solving the problem of optimal operation of multi-reservoir systems in order to supply the downstream demand.

**Figure 12:**
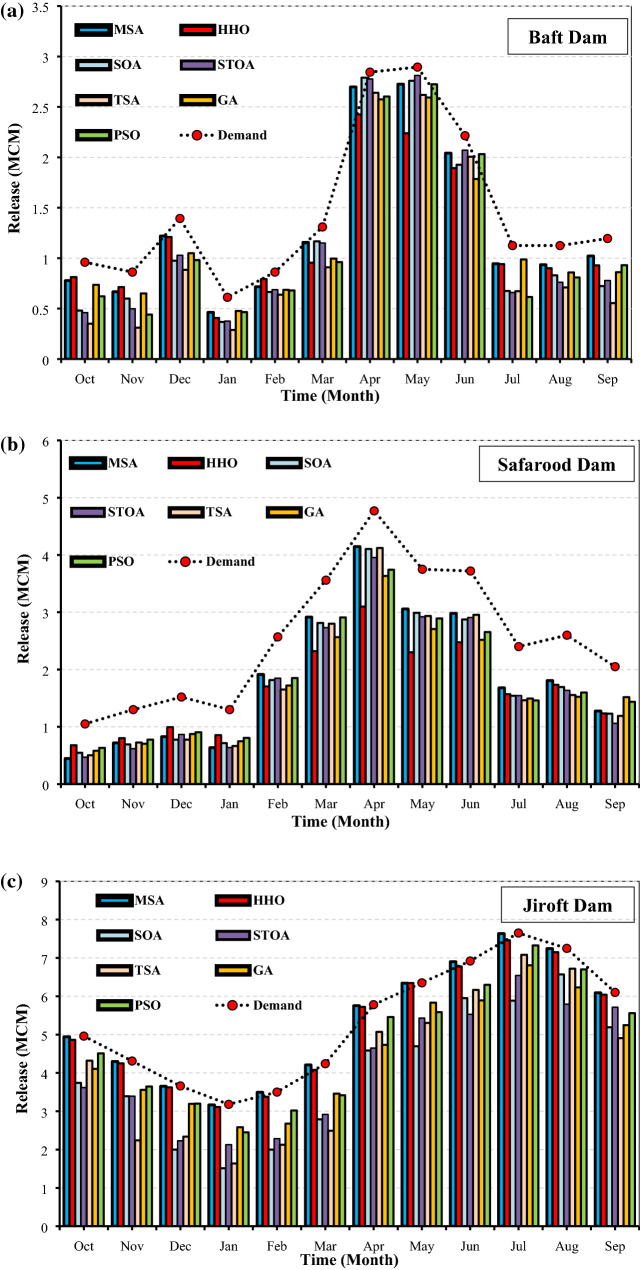
(**a**) Average monthly release and demand of Baft reservoir. (**b**) Average monthly release and demand of Safarood reservoir. (**c**) Average monthly release and demand of Jiroft reservoir.

Figure 12a–c show the optimized average monthly water release pattern from the Baft, Safarood and Jiroft reservoirs simulated by the studied algorithms during the study period (2000–2019). As can be seen, the optimized values of release by the MSA algorithm have a better estimate of the downstream demands for all the studied reservoirs, and it resulted in the minimum deficit in the water supply. The MSA-based release policy in Baft, Safarood, and Jiroft reservoirs was able to supply the downstream demands, during the 19-years of operation, by the reliability of 74%, 49% and, 99%, respectively.

**Figures 13:**
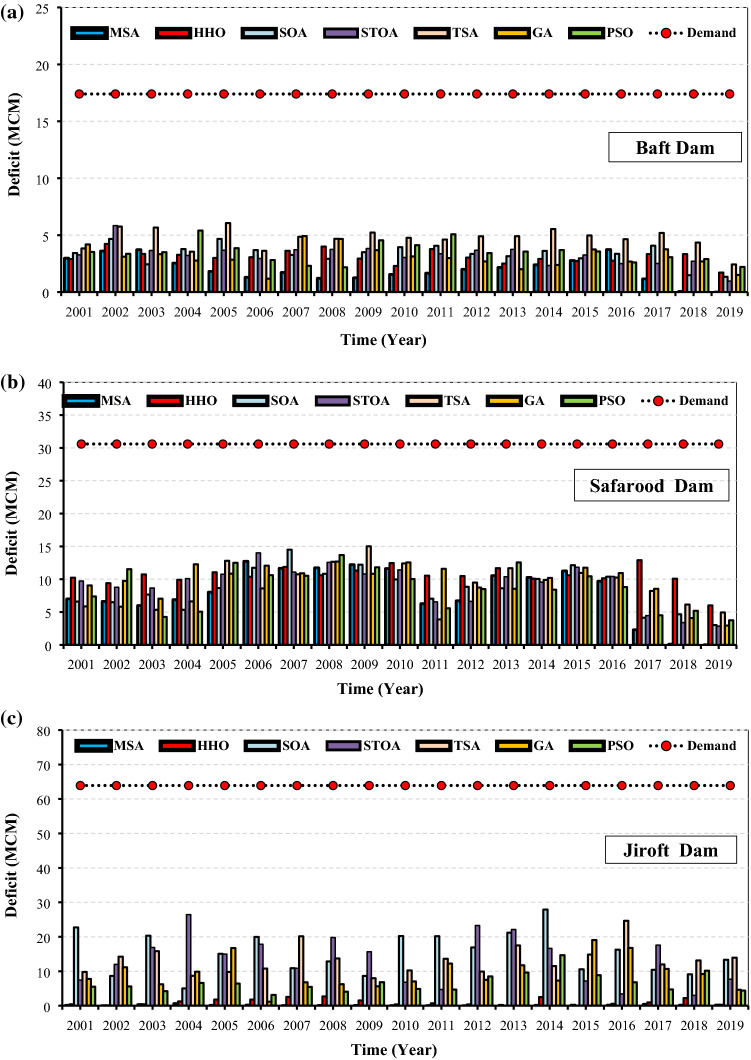
(**a**) Total annual deficit of Baft reservoir. (**b**) Total annual deficit of Safarood reservoir. (**c**) Total annual deficit of Jiroft reservoir.

Figure 13a–c show the total annual deficit in the Halilrood multi-reservoir system optimized by the studied algorithms. As can be seen, the MSA algorithm was well capable of minimizing the deficiencies of Baft, Safarood, and Jiroft reservoirs by 38, 151, and 3 million cubic meters, respectively, over 123 months of operation. The MSA-based deficit values had more appropriate estimates of the deficits for all the studied reservoirs, i.e., it demonstrated a high ability to supply demand and reduce the system’s deficit. The results of Fig. 13a–c are consistent with those of Fig. 12a–c, in which, the MSA is the best and SOA is the worst algorithm in solving large-scale problems.

## Conclusion

Developing optimal operation policies for single or multi-reservoir systems is a complex engineering problem. Solving this complex problem requires a robust, and high-performance method. In this study, four new-introduced evolutionary algorithms of HHO^19^, SOA^20^, STOA^21^, TSA^18^ and MSA^22^ were developed for the optimal operation of a multi-reservoir system. The performance of these algorithms in the optimization of Halilrood multi-reservoir operation was compared with each other, by four criteria of reliability, resilience, vulnerability, and sustainability. The results showed that the MSA with the objective function of 6.96 has a better performance compared to the HHO (14.68), SOA (31.28), STOA (29.55), TSA (25.32), GA (21.26), and PSO (16.04). Another aspect of the superiority of the MSA algorithm over the other four algorithms was the computational speed (CPU run time); it was 6739 s for MSA, 9347 s for PSO, 10,223 s for HHO, 11,782 s for GA, 12,971 s for SOA, 13,763 s for STOA, and 14,416 s for TSA. The demand deficit for the Baft, Safarood, and Jiroft reservoirs were equal to 37.66, 151.68, and 2.99 million cubic meters by the MSA algorithm, while, the corresponding values for the HHO algorithm, which had the second rank among the utilized algorithms, was 58.78, 200.46, and 20.52 million cubic meters, respectively. Finally, it was shown that the MSA-based optimized operating policy for the Halilrood multi-reservoir system, with sustainability criteria of 57.87 (Baft reservoir), 37.55 (Safarood reservoir), and 86.09 (Jiroft reservoir), demonstrated the highest performance among the utilized algorithms. Therefore, the use of MSA algorithm to extracting the operation policies of complex multi-reservoir systems is strongly recommended. In addition, the dynamic simulation of multi-reservoir systems by other robust optimization algorithms is recommended for future works.

## Data Availability

All data, models, or code generated or used during the study are available from the corresponding author by request.

## List of symbols

*F(Re)*: Objective function
*Re*_*i,t*_: Release from the ith reservoir during period t (MCM)
*De*_*i,t*_: Downstream demands of the ith reservoir during period t (MCM)
*De*_*i,Max*_: Maximum downstream demand of the ith reservoir in the whole operation period (MCM)
*i*: Reservoir index
*T*: Total number of operation periods (month)
*Penalty*: Penalty function
*Sp*_*i,t*_: Overflow from the ith reservoir during period t (MCM)
*S*_*i,t*_: Storages of the ith reservoir at the beginning of period t (MCM)
*S*_*i,t*__+__*1*_: Storages of the ith reservoir at the end of period t (MCM)
*S*_*max i*_: Maximum storage in the ith reservoir during period t
*S*_*min i*_: Minimum storage of the ith reservoir at the beginning of period t
*Q*_*i,t*_: Inflow into the ith reservoir during period t (MCM)
*Loss*_*i,t*_: Evaporation loss from the ith reservoir surface during period t (MCM)
*Ev*_*i,t*_: Net evaporation (evaporation minus precipitation) from the ith reservoir during the period t (m)
*a*_*i*_*, b*_*i*_* and c*_*i*_: Coefficients of storage-area relation for reservoir i
*A*_*i,t*_: Area of the ith reservoir during period t (KM^2^)
*Dez*_*i,t*_: Environmental demand of ith reservoir during period t (MCM)
*Re*_*max i,t*_: Maximum allowable release from the ith reservoir during period t
*Re*_*min i,t*_: Minimum allowable release from the ith reservoir during period t
*Rel*: Reliability index
*Val*: Vulnerability index
*Res*: Resiliency index
*SI*: Sustainability index
*NDe*_*f*_: Total number of failures occurred during the operation period
*De*_*t*_: Demand in period t
*Re*_*t*_: Release value of period t
*Def*_*t*_: Water deficit in period t
*Def*_*t*__+__*1*_: Water deficit in period t + 1

## Abbreviations

*MCM*: Million cubic meter
LP: Linear programming
NLP: Non-linear programming
DP: Dynamic programming
SDP: Stochastic dynamic programming
EA: Evolutionary algorithm
HHO: Harris Hawks optimization
SOA: Seagull optimization algorithm
STOA: Sooty tern optimization algorithm
TSA: Tunicate swarm algorithm
MSA: Moth swarm algorithm
GA: Genetic algorithm
PSO: Particle swarm optimization
WCA: Water cycle algorithm
HS: Harmony search
ICA: Imperialist competition algorithm
ANN: Artificial neural network
BA: Bat algorithm
SCE: Shuffled complex evolution
WSA: Wolf search algorithm
WOA: Whale optimization algorithm
MOMPC: Multi-objective model predictive control
